# Acute Heart Failure Caused by Parvovirus B-19 Myocarditis Treated with Human Immunoglobulin

**DOI:** 10.1155/2012/180871

**Published:** 2012-07-10

**Authors:** Luca Alberti, Marco Loffi, Gabriele Fragasso, Roberto Spoladore, Carlo Ballarotto, Alberto Margonato

**Affiliations:** ^1^Heart Failure Unit, Ospedale San Raffaele, Via Olgettina 60, 20132 Milano, Italy; ^2^Coronary Care Unit, Ospedale San Raffaele, Via Olgettina 60, 20132 Milano, Italy

## Abstract

We describe the case of a 42-year-old woman developing cardiogenic shock with cardiac tamponade due to an acute myo-pericarditis caused by Parvovirus B19 (PVB19), successfully treated with intravenous (iv) immunoglobulin administration.

## 1. Case Report

A 42-year-old healthy woman went to the emergency department after a malaise episode with fever, abdominal pain, and vomiting for few days. Past medical history was silent with no cardiovascular risk factors. Physical examination revealed a blood pressure (BP) of 70/50 mmHg, heart rate was 90 bpm, temperature 37.5°C, Sat O_2_ 98%.

Cardiopulmonary examination was normal, without any additional sounds, murmurs, or pericardial rub and any pulmonary crackles. There was also neither jugular turgor nor peripheral oedemas. The ECG performed in the emergency department showed sinus tachycardia with diffuse low voltages.

Laboratory investigations revealed high levels of white blood cell (WBC) count 18200/mm^3^, C-reactive protein (CRP) 2.93 mg/dL, haemoglobin 10.8, INR 1.3, and antithrombin III 57%. The first set of cardiac enzymes was elevated CKMB 7,3 and Troponin-T1.13. Haemogas analysis showed metabolic acidosis pattern with severe hypoxia (pH 7.31, pCO_2_ 42, pO_2_ 33, HCO_3_^−^ 21, BE 5). At chest X-ray neither, signs of pleural effusion nor lung consolidations were present. Transthoracic echocardiography showed a moderate pericardial effusion, left ventricular hypokinesis with ejection fraction of 35%. Abdominal ultrasound scan revealed no free fluid or biliary tract dilatation. There was a rapid worsening of clinical conditions complicated by cardiac arrest, which was successfully treated by cardiopulmonary resuscitation procedures. Dopamine at 10 *μ*g/Kg/min was started. A chest computerized tomography scan (CT) excluded pulmonary thromboembolism and an abdominal CT excluded sources of haemorrhage. Another hypotensive episode with bradycardia occurred, epinephrine 0,25 *μ*g/kg/min was introduced and dopamine was reduced to 4,5 *μ*g/Kg/min. The patient was then transferred to our post cardiac surgery intensive care unit. On physical examination performed at admission, her BP was 90/60, despite inotropic support with epinephrine and dopamine; the heart rate was 130 bpm and on ECG tracing several run of atrial fibrillation with elevated ventricular response were registered. Central venous pressure (CVP) was 20 mmHg. Haemogas analysis showed metabolic acidosis pattern. A laboratory investigation revealed the following data: WBC 23700/*μ*L; CRP 37.7 mg/dL; glucose 302 mg/dL; N-terminal-pro brain natriuretic peptide (NT-proBNP) 12650 pg/mL: troponin-T 0.082. Transthoracic and transesophageal echocardiographies were performed with evidence of biventricular failure (EF < 20%, TAPSE 10); 2 cm pericardial effusion with initial signs of tamponade (complete right atrium and partial right ventricle collapse); moderate mitral regurgitation: mild tricuspidalic regurgitation. Epinephrine was increased to 0.3 *μ*g/kg/min. A pericardiocentesis was performed, but drainage of 150 mL of sierous, citrine liquid resulted only in partial improvement in global systolic function (EF 25%, BP 70/40 mmHg, CVP 8 mmHg, wedge pressure 15 mmHg, PAPs 30 mmHg). Dopamine was stopped and the patient was transferred to Cardiac Cath Lab where coronarography showed normal coronary vessels. Subsequently, an endomyocardial biopsy was performed showing, by the PCR analysis, positivity for Parvovirus B19 genome at high title (1200 UL/mL). The histological and immunohistochemistry analysis of myocardial tissue revealed focal myocytes vacuolization, mild fibrosis, and moderate lymphocytes infiltrate both T (CD3+), and B (CD20+), rare plasma-cells (CD138+) and macrophage monocytes (CD68+). The histological pattern was reliable with myocarditis. Intra-aortic balloon pump (IABP) was placed with modest improvement in mean arterial blood pressure (88 mmHg). According to the endomyocardial biopsy results, i.v. high doses pentaglobulin treatment was started. Some hours after IABP placement and immunoglobulins administration, transthoracic echocardiography showed improvement in biventricular contractile function (cardiac index 2.4 mL/min*·*m^2^). Another pericardiocentesis was performed and 180 mL of sierohaematic liquid was drained. The following day, epinephrine was reduced to 0.16 *μ*g/kg/min and norepinephrine 0.05 *μ*g/kg/min was introduced. Myocardial systolic function gradually improved (EF 55–60%, cardiac index 3.5 mL/min*·*m^2^). Epinephrine and norepinephrine dosage was gradually reduced and then stopped. IABP was then removed. Clinical conditions were stable so the patient was extubated and transferred to coronary intensive care unit. On clinical examination at admission, her BP was 135/85 mmHg with sinus tachycardia (135 bpm).

Laboratory investigations showed an increase of WBC count of 44000/mm^3^ (94% neutrophils), haemoglobin 9.2, platelet count of 85000/mm^3^, CK-MB 7575 (MB 66), troponin-T 0.155, LDH 549, NT-proBNP 24000. Complete autoantibodies screening (ANA, aDNA, SSA, SSB, SM, RNP, Scl70, Jo-1) was performed but revealed no evidence of any autoimmune disorders; a screening for common pathogens involved in myocarditis was performed too. There were not antibodies anticoxsackie or echo virus. Polymerase chain reaction (PCR) was made on plasma samples looking for the genome of: EBV, CMV, HSV 1, HSV 2, HHV 6, adenovirus, enterovirus, echovirus with negative results. A high title (>1 : 40) of IgM antiparvovirus B19 was found. Haemocultures and pericardial fluid cultures did not show any growth. In the following days, there was reduction of cardiac and inflammatory enzymes. Echocardiography showed normal systolic left and right ventricular function, moderate tricuspid regurgitation, PAPs of 35 mmHg, no pericardial fluid. Rate control therapy was started with bisoprolol 1.25 mg qd. Normalization of cardiac and inflammatory enzymes was observed, the patient gradually recovered and she was discharged from our hospital.

## 2. Discussion

This case illustrates PVB19-induced myopericarditis leading to severe cardiac dysfunction. Parvovirus B19 infection could lead to direct myocardial injury, and could trigger a severe autoimmune reaction of the myocardium [1]. Myocarditis is an inflammatory disease with a variable natural history, ranging from spontaneous recovery, subclinical course with evolution to dilated cardiomyopathy (DCM) or early death from multisystem failure [2, 3]. The progression to DCM can result from both direct viral-mediated damage or secondary autoimmune-mediated myocardial injury [4]. This secondary autoimmunity is induced by molecular mimicry, inducing cross-reaction between antiviral antibodies with autoantigen-binding properties, a phenomenon demonstrated also in parvovirus B19 myocardial infections [5, 6]. In a previous study by Dennert et al., I was shown how immunoglobulin therapy in addition to conventional heart failure therapy is able to improve cardiac function in patients with chronic dilated cardiomyopathy (DCM) and a significant PVB19 viral load in the heart [7]. Based on these evidences, we tried to perform the same treatment modality obtaining an early improvement in left ventricular performance. The peculiarity of this case is the effectiveness of immunoglobulin administration in the acute phase of heart failure. According to this result, it is now time to intensify the diagnostic tools in order to get an early etiologic diagnosis of myocarditis and to perform dedicated treatment options that could promptly restore an acute cardiac impairment, blocking the possible evolution towards end-stage forms of dilated cardiomyopathy. 
